# Rescue Cervical Cerclage After 24 Weeks and Subsequent Childbirth After 34 Weeks of Gestation

**DOI:** 10.7759/cureus.58274

**Published:** 2024-04-14

**Authors:** Dionysios G Galatis, Christos Benekos, Panagiotis-Konstantinos Karachalios, Konstantina Kalaitzi, Vasileios Batsakoutsas, Ioannis Chatzipanagiotis, Ippokratis Diamantakis, Argyrios Monastiriotis, Nikolaos Kiriakopoulos

**Affiliations:** 1 Obstetrics and Gynecology, National and Kapodistrian University of Athens School of Medicine, Athens, GRC; 2 V’ Department of Obstetrics/Gynecology, Helena Venizelou General and Maternity Hospital, Athens, GRC

**Keywords:** preterm membrane rupture, rescue cerclage, cervical cerclage, cervical incompetence, cervical insufficiency

## Abstract

A defect in the structure or function of the cervix that causes it to fail to contain the fetus intrauterine creates the condition called cervical insufficiency. Typical symptoms are pressure in the area of the pelvis, premature membrane rupture, and cervical dilation without uterine contractions. Surgical treatment includes the technique of cervical cerclage. It is usually performed from week 12 to week 16 of pregnancy. This article presents a case of rescue cervical cerclage after 24 weeks gestation and the observance of the pregnancy that followed. The cerclage was successful in prolonging the gestation of the fetus and no post-operative complications occurred due to the operation. The outcome of the pregnancy was a live and healthy baby born at 34 weeks gestation.

## Introduction

A defect in the structure or function of the cervix that causes it to fail to contain the fetus intrauterine creates the condition called cervical insufficiency. The cervix ripens and dilates without being accompanied by contractions of the uterus. Various infections of the lower genital tract are the most common trigger of premature cervical dilation [1]. Typical symptoms that patients are presented with are pressure in the area of the pelvis, premature membrane rupture in the first two trimesters, and cervical dilation without uterine contractions. The occurrence rate of cervical insufficiency 0.5% to 1% and risk of reappearance can reach as high as 30% [2].

Treatment of cervical insufficiency has been approached from multiple angles by clinicians. Nonsurgical methods, such as bed restriction, have been found inadequate to prevent the relapse of the condition [1]. Surgical treatment includes the technique of cervical cerclage. This technique places stitches above the opening of the cervix with the goal of reinforcing the muscle tissue of the cervix and tightening the opening of the cervical canal. It is usually performed in week 12 to week 16 of pregnancy [3]. This article presents a case of rescue cervical cerclage after 24 weeks gestation and the observance of the pregnancy that followed.

## Case presentation

The patient, 28 years old, G1P1, pregnant at 24+4 weeks' gestation, presented in the emergency department of the hospital, reporting feelings of bloating and heaviness in the lower region of her reproductive organs. She did not report abdominal pain or any other signs of going into labour. Her obstetric history did not include any miscarriages or gynaecologic surgeries. Routine examinations during her antenatal care were normal, with no history of previous urinary or vaginal infections. A B-level scan was performed at 20+3 weeks’ gestation and found no abnormalities. During the scan, the length of the cervix was found to be 26.0 mm.

**Figure 1:**
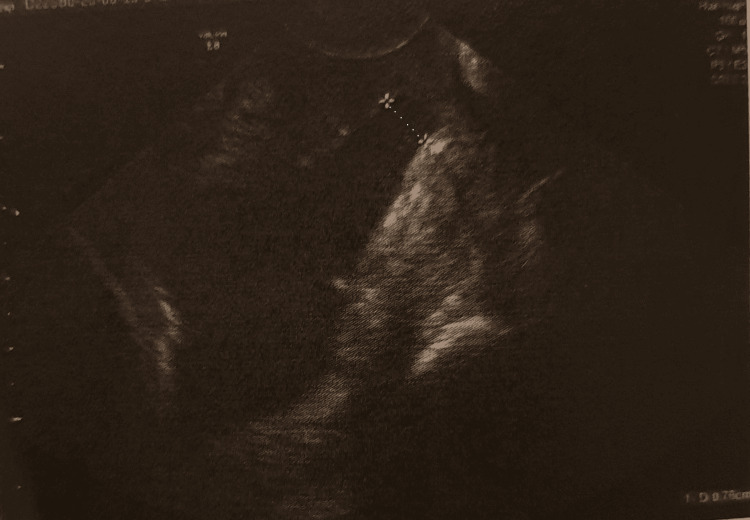
Ultrasound image The image shows cervical dilation at 7.6 mm.

Basic clinical signs were measured and found between normal parameters (blood pressure 122/68 mmHg, heart rate 75 beats per minute (bpm), temperature 36.6°C). The cardiologic examination was normal. The height of the uterine fundus was according to the gestational age of the pregnancy. During vaginal examination, the cervix was presented with near complete effacement, dilation of 7.6 mm, and a discernible bulging amniotic sac (Figure 1). The fetal fibronectin test was found to be negative. Ultrasound scan presented a single live fetus 24 weeks gestation, with an approximate fetal weight of 691 g.

The patient was admitted to the hospital with the diagnosis of G1P1, 24+4 weeks’ gestation, with cervical incompetence. Blood tests and serological examinations performed for common infections were found normal. The vaginal culture was taken and found free of infections. Betamethasone doses were administered. Bed confinement was instructed.

In order to prevent preterm labour with possibly fatal results for the fetus, a rescue cervical cerclage by McDonald procedure was planned under general anesthesia. The patient was placed in the dorsal lithotomy position. After surgical preparation of the vagina and perineum, the bladder was drained. An inflated foley balloon was used to reposition the amniotic membranes. Continuous stitches were applied with a no. 2 monofilament suture at positions 12, 3, 6, and 9 o’clock. After encirclement, the suture was tightened and tied.

Post-operatively, broad-spectrum antibiotics (amoxicillin 1 g x 2 for 7 days) and progesterone were administered. Daily blood tests for infection and frequent fetal heartbeat monitoring were performed. A week later, the patient showed no post-operative complications. At 28 weeks gestation, the patient reported abdominal pain and was administered tocolytic treatment with atociban. The next day the pain subsided. At 34 weeks gestation, there was a preterm rupture of membranes and the patient went into labour. Due to the severity of the incident, an urgent caesarean section was decided. A live and healthy male baby, 2680 g, was delivered with an Apgar score of 9 at the first minute and 10 at the fifth minute. The neonate was sent to an Intensive Care Unit and was discharged five days later.

## Discussion

It is currently recommended to perform a cervical cerclage on patients with an obstetric history of spontaneous preterm birth or a previous diagnosis of cervical incompetence and have a cervix with a length less than 25 mm in the current pregnancy [4]. Serial measurements of the cervix length can be used under ultrasound imaging in order to avoid performing cerclage on women that would have minimal effect [5]. The effects of cervical cerclage on women who present bulging membranes and cervical insufficiency have been well documented. The use of this technique in women with cervical incompetence can lower the rate of preterm delivery compared to expectant management. In addition, performing cervical cerclage in populations at high risk of preterm birth has the effect of significantly lowering perinatal morbidity and mortality [6]. The survival rate of the fetus of an expectant mother with rescue cervical cerclage is reported to be between 48% and 68% [3].

Rescue cervical cerclage is a controversial technique, whose advantages are still debated in official literature. While it can be used to further the pregnancy to ideally viable weeks of gestation and concur to a positive outcome of pregnancy, exposing the membranes of the fetus to the vaginal flora carries the risk of infection of the amniotic sac and the fetus [7]. In our case, we performed an emergency rescue cerclage on a patient pregnant at 24+4 weeks’ gestation with the diagnosis of cervical insufficiency. The cerclage was successful in prolonging the gestation of the fetus and no post-operative complications occurred due to the operation. The outcome of the pregnancy was a live and healthy baby born at 34 weeks gestation.

## Conclusions

Cervical insufficiency is a condition that can lead to preterm delivery or worse, to miscarriage. Rescue cerclage is a procedure that, while it carries risks, can aid in the prolonging of the gestation to a viable age. There is a debate as to when it is preferred to be performed regarding the gestation age; however, as shown in this case, there is certain merit in performing it after 24 weeks, lending the fetus as much time as possible for safe growth and maturation. More research regarding this issue could aid in the treatment of future cases of cervical insufficiency.
